# Macrophages From Latently Infected Mice Have Trained Immunity to HSV-1

**DOI:** 10.1167/iovs.67.2.49

**Published:** 2026-02-24

**Authors:** Ujjaldeep Jaggi, Shaohui Wang, Satoshi Hirose, Deepak Arya, Homayon Ghiasi

**Affiliations:** 1 Department of Surgery, Center for Neurobiology and Vaccine Development, Ophthalmology Research, Cedars-Sinai Health Science University, Los Angeles, California, United States

**Keywords:** macrophages, ocular infection, herpes simplex virus 1 (HSV-1), training, memory

## Abstract

**Purpose:**

Our previous studies demonstrated that macrophages play a crucial role in both primary and latent herpes simplex virus 1 (HSV-1) infections. Here, we sought to determine whether HSV-1 exposure induces long-lasting functional and epigenetic changes in macrophages consistent with trained immunity, leading to enhanced responses upon secondary stimulation.

**Methods:**

To explore this, we performed Assay for Transposase-Accessible Chromatin sequencing (ATAC-seq) analysis on isolated spleen- and bone marrow (BM)-derived macrophages from latently infected mice before and after stimulation with UV-inactivated virus to identify open chromatin regions indicative of changes in gene regulation. Additionally, we performed flow cytometric analysis of infected spleen macrophages, BM-derived macrophages, corneas, and the trigeminal ganglia (TG). Moreover, to assess the durability of training response to infection, we evaluated responses after secondary infection.

**Results:**

The study revealed that immunity-related GTPase family M protein (IRGM1) expression in isolated macrophages from latently infected mice was significantly elevated after stimulation, compared with that of more than 900 genes with open or closed chromatin accessibility. Flow cytometry further confirmed a higher proportion of IRGM1^+^ macrophages in the spleen, BM, cornea, and the TG of latently infected mice compared with mock-infected controls. The qRT-PCR determined that macrophages isolated from the spleen, trigeminal ganglia, and BM of latently infected mice continued to exhibit elevated IRGM1 expression levels relative to controls.

**Conclusions:**

Collectively, our findings indicate that macrophages develop a durable trained immunity to HSV-1 infection, with IRGM1 emerging as a key component in the long-term maintenance of macrophage immunological memory.

Herpes stromal keratitis (HSK), caused by herpes simplex virus 1 (HSV-1), is broadly categorized into two stages. The preclinical stage involves the rapid infiltration of innate immune cells that aid in virus clearance from the eye, after which the virus becomes latent in the trigeminal ganglia (TG).^1^^,^^2^ The clinical stage is characterized by the involvement of adaptive immune cells, which drive immunopathology and initiate periodic episodes of virus reactivation.^3^ Understanding the role of innate immunity in HSK is critical, as it shapes and initiates subsequent adaptive immune responses. One of the key cell infiltrates in HSV-1 infection, as established by our group, is the macrophages, which infiltrates the infected eye as early as 1 hour post infection (PI).^4^ Macrophages are widely distributed throughout the body and display a unique transcriptional profile in each setting.^5^^–^^7^ They play key roles in immune defense mechanisms, including central roles in innate or natural immunity.^8^^–^^11^ They are also critical for the balance and efficacy of the immune responses, representing an interface between innate and adaptive immunity.^12^ As macrophages exhibit a wide variety of functions, including phagocytosis, cytokine secretion, antigen presentation, and tumor cytotoxicity.^13^^,^^14^ They appear to be very important in both exacerbating and controlling acute and chronic HSV-1 infection.^15^^–^^23^ Thus, identifying the roles of macrophages in ocular infection requires rigorous and comprehensive analyses of macrophage responses to infection. Our previous work has demonstrated two key findings regarding macrophage involvement in HSV-1 ocular infection: (1) macrophages are one of the most abundant and dominant infiltrates into the mouse corneas after ocular infection^4^; and (2) macrophage depletion increases virus replication in both the eyes and the TG of infected mice, underscoring their protective role in early antiviral defense.^19^

The innate immune memory of macrophages has gained importance with the emergence of immune memory in tissue-resident macrophages in vivo, as demonstrated by epigenetic analyses.^12^^,^^24^ In bacterial infections, alveolar macrophages have exhibited robust and critical memory responses.^25^ Macrophages also play a key role in the protective memory induced by the BCG vaccine against *Mycobacterium tuberculosis* (*M. tuberculosis*) infection.^26^ Despite these advances, the potential for macrophages to develop immunological memory in response to viral infections such as HSV-1 remains largely unexplored. To examine the timing and nature of possible immunological memory to HSV-1, we performed an Assay for Transposase-Accessible Chromatin with high-throughput sequencing (ATAC-seq). This genome-wide method allows us to identify regions of open chromatin, providing insights into the regulatory landscape of immune cells during memory formation.^27^ Using this method, we conducted a comprehensive analysis of epigenetic and transcriptional changes in macrophages isolated from the spleen, BM, TG, and corneas of latently infected mice. The goal was to uncover molecular signatures associated with potential trained immunity.

Our ATAC-seq analysis identified more than 900 genes with chromatin accessibility changes, encompassing both open and closed chromatin regions. Among these, IRGM1 emerged as a gene of particular interest due to its significantly higher accessibility peaks in macrophages isolated from the spleen and BM of latently infected mice. IRGM1 is a member of the Immunity-related GTPases (IRG) family and regulates membrane remodeling events in cells, including autophagy and mitophagy.^28^ Ras signaling is known to be regulated by GTPase activity,^29^ despite IRGM1 being a member of GTPases, but IRGM1 is not known to directly regulate Ras signaling. Other studies reported IRGM1 in macrophages supports memory responses by maintaining mitochondrial integrity during activation, regulating autophagy-dependent epigenetic changes, and modulating inflammatory cytokine production (e.g., TNF-α and IL-6).^30^ Furthermore, single-cell analysis of macrophages from latently infected TGs also showed upregulation of IRGM1. Upon stimulation with UV-inactivated HSV-1, BM-derived macrophages exhibited a pronounced increase in chromatin accessibility at the IRGM1 locus compared with all other genes with open chromatin regions, underscoring IRGM1’s fundamental role in the macrophage memory or trained immunity response to HSV-1. To study the duration of memory response against HSV-1, we performed qRT-PCR analysis of IRGM1 expression in BM-derived macrophages, isolated spleen macrophages, and total TG tissue at day 70 PI, extending beyond the previously studied day 35 PI. Our data demonstrate that the IRGM1-associated memory response to HSV-1 persists at least until day 70 PI.

Taken together, these findings indicate that macrophages, similar to CD4⁺ and CD8⁺ T cells, natural killer (NK) cells, dendritic cells (DCs), group 2 innate lymphoid cells (ILC2s), and B cells, develop a form of immunological memory (trained immunity) to HSV-1 infection. This macrophage memory associated with IRGM1 expression likely contributes to enhanced protection against ocular HSV-1 infection.

## Materials and Methods

### Mice and Viruses

Six- to 7-week-old female C57BL/6 mice were used for the experiments and purchased from the Jackson Laboratory (Bar Harbor, ME, USA). Mice were infected ocularly with 2 × 10^5^ pfu/eye of Plaque-purified, virulent HSV-1 strain McKrae or mock control without corneal scarification. Mock infected control mice were ocularly treated with media only. All procedures were performed in strict accordance with the Association for Research in Vision and Ophthalmology Statement for the Use of Animals in Ophthalmic and Vision Research and the NIH Guide for the Care and Use of Laboratory Animals (ISBN 0-309-05377-3). The animal research protocol was approved on October 17, 2021, by the Institutional Animal Care and Use Committee of Cedars-Sinai Medical Center (protocol no. 8837).

### ATAC-Seq Library Preparation and Sequencing

On day 35 PI, BM and spleens from infected and mock infected mice were isolated and differentiated into macrophages as described below. Spleens were sorted for macrophages as described below. BM-derived macrophages were stimulated with UV-inactivated virus, and 24 hours post-stimulation, cells were harvested and subjected to ATAC-seq performed by the Applied Genomics, Computation, and Translational Core at Cedars-Sinai Medical Center, Los Angeles, California, USA.

Briefly, suspensions of 50,000 cells with viability >90% were processed for ATAC-seq according to the Omni-ATAC-Seq protocol by Corces et al.^31^ Cell suspensions were centrifuged, supernatant removed, and then cells were lysed on ice for 3 minutes. Nuclei were then centrifuged, and the supernatant removed. Transposition master mix containing Tagment DNA TDE1 Enzyme (Illumina, San Diego, CA, USA) was added to simultaneously fragment and add Illumina adapter sequences to open chromatin. After transposed chromatin cleanup, unique single-indexes were added to the DNA fragments in a PCR reaction to generate Illumina sequencing libraries. Library concentration was measured via the Qubit 1X dsDNA HS Assay kit, and library size on the Agilent 4200 TapeStation (Agilent Technologies) with the Agilent HS D5000 ScreenTape. Multiplexed libraries were pooled and sequenced on a NovaSeq 6000 or NextSeq 500 (Illumina) using 1 × 75 bp sequencing at approximately 40 M reads/sample.

### Preparation and Stimulation of Macrophages From BM

Femoral bones were dissected, and each bone end was cut off, and the BM was expelled. BM cells were cultured for 6 days and were further differentiated and activated as we described previously.^32^^,^^33^ BM-derived macrophages described above were seeded at 2 × 10^5^ cells/well in a 24-well plate, and, on the following day, the cells were stimulated with 10 pfu/cell of UV-inactivated HSV-1 strain McKrae for 1 hour. Twenty-four hours post-stimulation, the cells were washed three times with PBS, and fresh complete DMEM was added to each well. Infected cell monolayers were harvested and subjected to flow cytometry, ATAC-seq, and RT-PCR.

### Single-Cell Analysis of Cells Isolated From the TG of Latently Infected Mice

As we described previously,^34^ on day 35 PI, latently infected mice or mock infected controls were terminated, and the TGs were harvested. Briefly, CD45^+^ single cells were isolated, and libraries were sequenced on a NovaSeq 6000 (Illumina, San Diego, CA, USA) according to the Single Cell 3′ version 3.1 Reagent Kits User Guide, with a sequencing depth of approximately 40,000 reads per cell. The ImmGen reference was used to determine the macrophage cell type.

### Flow Cytometry and Cell Sorting Analysis

Flow cytometry and cell sorting analysis were performed on BM-derived macrophages, corneas, and spleen macrophages from infected and mock infected mice, as described below, for each tissue. Flow cytometry was performed on a BD Symphony A5 Cell Analyzer, and analysis was conducted using FlowJo software, whereas cell sorting was done using a BD Aria 3 sorter.

A. Spleen macrophages sorting and staining. Spleens were harvested from infected and mock infected mice on day 35 PI, cut into small pieces, and digested with collagenase D (2 mg/mL) and DNase I (20 U/mL) in 1 × HBSS at 37°C for 30 minutes. Single-cell suspensions were prepared by mechanically dissociating the digested spleens through a cell strainer. Red blood cells were removed by incubating the cells with 1 × RBC lysis buffer (Sigma) for 3 minutes at room temperature. Briefly, the cells were washed with cold 1 × PBS, blocked with TruStain FcX (BioLegend) in 1 × staining buffer (BioLegend, #420201), and then stained with the indicated antibodies, according to the manufacturer's protocols (BioLegend). Antibodies used for spleen macrophage sorting were APC-CD11b (BioLegend, #101212), PE-F4/80 (BioLegend, #123110), FITC-CD3 (BioLegend, #100204), FITC-Ly6G (BioLegend, #127606), and FITC-CD19 (BioLegend, #115506). For sorting, we used CD45, F4/80, and Alexa Fluor 647–conjugated anti-IRGM1 (Novus, #76377AF647). For IRGM1 staining, cells were first stained with surface antibodies, then fixed and permeabilized, and intracellularly stained with an anti-IRGM1 antibody, according to the manufacturer’s protocol. Experiments were also conducted on isolated spleen cells from infected and mock infected mice, and single cells were generated. These cells were then stained with antibodies targeting CD45^+^ and F4/80^+^ markers. Stained cells were sorted for macrophages and used for RT-PCR analysis.
B. Sorting of BM-derived macrophages. Cells from the BM of infected and mock infected mice were counted and plated with macrophage stimulatory growth factors as described above. On day 6 PI, macrophages were scraped out, counted, plated, and further stimulated with the UV-inactivated HSV-1 strain McKrae (10 pfu/cell) for 1 hour, and then harvested at 24 hours post-stimulation. Cells were stained with indicated antibodies to PE-F4/80, BV421-CD45, and lineage markers FITC-CD3, FITC-CD19, FITC-Ly6g, and 7-AAD. Stained cells were analyzed by BD Symphony A5 Cell Analyzer. Similar to spleen-sorted macrophages (described above), BM-derived macrophages were also used for RT-PCR analysis.
C. Sorting and staining corneas. Corneas were harvested from both infected and mock infected mice. For each group, the corneas were pooled, placed in ice-cold RPMI containing 10% FBS, and finely minced. Tissue was enzymatically digested using 1.25 mg/mL Liberase (Roche) and RNase-free DNase (Qiagen) at 37°C for 1 hour to generate single cell suspensions. Cells were blocked with Human TruStain FcX (BioLegend) at a concentration of one million cells to prevent nonspecific Fc receptor binding and subsequently stained with anti-CD45 and anti-F4/80 antibodies (BioLegend), according to the manufacturer's protocol. For intracellular IRGM1 detection, cells were fixed, permeabilized, and stained using an IRGM1-specific antibody following the manufacturer’s instructions. Data acquisition was performed on a BD Symphony A5 Cell Analyzer, and analysis was conducted using FlowJo software. CD45^+^F4/80^+^IRGM1^+^ macrophages were quantified and represented as histogram plots comparing infected and uninfected groups.

### RNA Extraction, cDNA Synthesis, and TaqMan RT-PCR

The BM, TG, corneas, and spleen were harvested on day 70 PI from infected and mock infected control mice. Sorted tissues were immersed in RNA stabilization reagent (RNA Later, Thermo Fisher Scientific, Waltham, MA, USA), and stored at −80°C until processing. Total RNA was extracted as described.^35^^,^^36^ One µg of total RNA for BM macrophages and the TGs, as well as 80 ng for spleen macrophages, was reverse transcribed using a high-capacity cDNA reverse transcription kit (Applied Biosystems, Foster City, CA, USA), according to the manufacturer's protocol. The mRNA expression levels of genes in the study were determined using: (1) IRGM1 (assay ID; Thermo Fisher, Mm00492595_m1; amplicon size, 84 bp) and (2) GAPDH (ABI Mm999999.15_G1—; amplicon length, 107 bp). GAPDH was used as a control in all experiments and to normalize transcript levels.

Quantitative real-time PCR (qRT-PCR) was performed using the TaqMan gene expression assay kit in 384-well plates on ABI QuantStudio 5 (Applied Biosystems, Foster City, CA, USA). Real-time PCR was performed in triplicate for each tissue sample. The threshold cycle (C_t_) values, representing the PCR cycle at which there is a noticeable increase in reporter fluorescence above baseline, were determined using SDS 2.2 software. To analyze fold change of expression, the 2−ΔΔCT method was used to calculate gene expression fold change compared to expression in uninfected controls in each group. Statistical difference was calculated by one-way ANOVA.

### Statistical Analysis

For all statistical tests, *P* values less than or equal to 0.05 were considered statistically significant and marked by a single asterisk (*). The *P* values less than or equal to 0.001 were marked by double asterisks (**). A two-tailed Student’s *t*-test with unequal variances was used to compare differences between two experimental groups. A 1-way ANOVA test was used to compare differences among three or more experimental groups. Graphs were plotted using GraphPad Prism 9 software. All experiments were repeated at least three times to ensure accuracy.

## Results

### ATAC Analysis Revealed That BM-Derived Macrophages From Latently Infected Mice Exhibit Increased Chromatin Accessibility at the IRGM1 Locus

**Figure 1.**
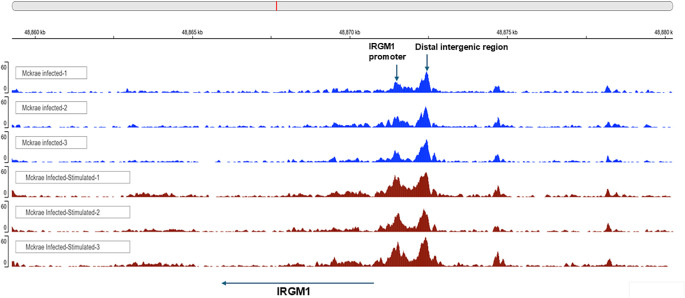
**Increased open chromatin near the IRGM1 promoter was observed in BM-derived macrophages from latently infected mice.** Mice were infected ocularly with 2 × 10^5^ pfu/eye of HSV-1. On day 35 PI, BM from infected mice was harvested, and macrophages were generated. On day 7 post differentiation, macrophages were stimulated for 24 hours with 10 pfu/cell of UV-inactivated HSV-1. BM-derived stimulated and unstimulated macrophages were stained with CD45, F4/80, and lineage (Lin) antibodies. The CD45^+^F4/80^+^Lin^−^ macrophages were sorted and subjected to ATAC-seq to assess chromatin accessibility. Peaks of open chromatin near the IRGM1 promoter were visualized using the IGV genome web browser (UCSD). The *arrow* indicates an increase in open chromatin near the IRGM1 promoter.

The action of transcription factors, along with regulatory elements such as promoters, enhancers, and repressors, tightly regulates gene expression. An additional level of protection is offered at the epigenetic level.^37^ One such standard method for studying epigenetic modifications and chromatin accessibility across the genome is ATAC-seq. This technique uses a hyperactive Tn5 transposase to cut genomic DNA and insert sequencing adapters into open chromatin regions. The DNA fragment with adapters can be PCR amplified and directly sequenced. Analyses have shown that ATAC-seq provides accurate, direct, and sensitive measurements of chromatin accessibility. However, the presence of macrophage memory in the context of viral infections, such as HSV-1, remains unknown. To decipher the timing and nature of possible immunological memory to HSV-1, we performed genome-wide analyses of epigenetic and transcriptional changes in a mouse model of ocular HSV-1 infection. Mice were infected ocularly with 2 × 10^5^ pfu/eye of HSV-1 strain McKrae with no scarification. On day 35 PI, BM were isolated, and macrophages were derived following the protocols described in Materials and Methods. To detect if stimulation can alter open chromatin levels of infected BM-derived macrophages, some of the BM-derived macrophages were stimulated with UV-inactivated HSV-1. After 24 hours post-stimulation, BM-derived macrophages were subjected to ATAC-seq. IRGM1 peak levels were elevated in stimulated BM-derived macrophages compared with unstimulated BM-derived macrophages (Fig. 1, arrow, IRGM1). The second peak detected in both stimulated and non-stimulated groups is located at the distal intergenic region to IRGM1 and, in contrast to the promoter region, this peak did not increase and may contain functional regulatory as well as junk elements (see Fig. 1, arrow, distal intergenic region). These findings suggest that the macrophage memory response to HSV-1 is associated with increased IRGM1 expression and is further enhanced upon antigenic stimulation. Therefore, our results indicate that macrophages develop memory of HSV-1 infection, and this memory is associated with higher IRGM1 expression.

### BM-Derived Macrophages From Latently Infected Mice Exhibit Higher IRGM1 Expression

**Figure 2.**
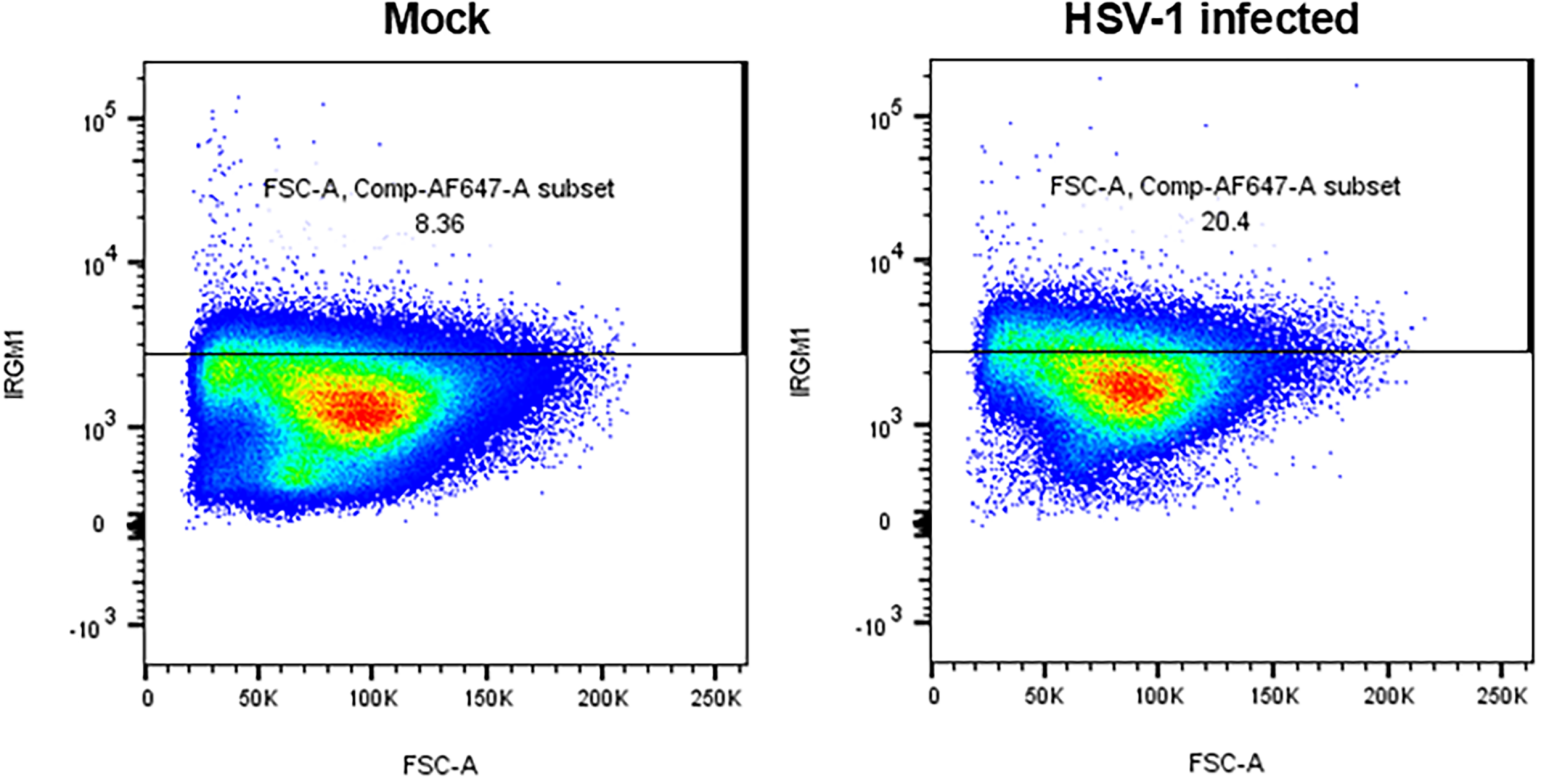
**Increased expression of IRGM1^+^ cells in BM-derived macrophages from latently infected mice.** Mice were infected ocularly with HSV-1 or mock infected as described above. On day 35 PI, BM was harvested, and macrophages were generated, as detailed in Materials and Methods. Cells were harvested and stained with the IRGM1-specific antibody. Percentages of IRGM1^+^ cells were evaluated by flow cytometry. Data is represented as percentages (%) of cells in each group.

To validate our ATAC-seq findings, which suggest increased chromatin accessibility at the IRGM1 locus, we evaluated IRGM1 expression in BM-derived macrophages from latently infected mice using flow cytometry. Mice were ocularly infected with 2 × 10^5^ pfu/eye of HSV-1 or mock infected. On day 35 PI, BM from latently infected or mock infected mice was harvested, and macrophages were generated and stimulated as described in Materials and Methods. After 24 hours post-stimulation, BM-derived macrophages were stained with antibodies against CD45, F4/80, CD3, CD19, Ly6g, and IRGM1 and analyzed by flow cytometry. The macrophages were gated for CD45^+^F4/80^+^CD3^−^CD19^−^Ly6g^−^IRGM1^+^ cells to look for IRGM1 expression in BM-derived macrophages after stimulation (Fig. 2). Analysis revealed that 20.4% of macrophages in the HSV-1-infected BM group were IRGM1⁺, compared to only 8.36% in the mock infected group (see Fig. 2). These results confirm our ATAC-seq findings and demonstrate that IRGM1 expression is significantly elevated in BM-derived macrophages from latently infected mice compared with mock infected control. This further supports the role of IRGM1 in macrophage-trained immunity to HSV-1 infection.

### Similar to BM-Derived Macrophages, Isolated Macrophages From the Spleens of Latently Infected Mice Also Showed Higher Chromatin Accessibility of IRGM1

**Figure 3.**
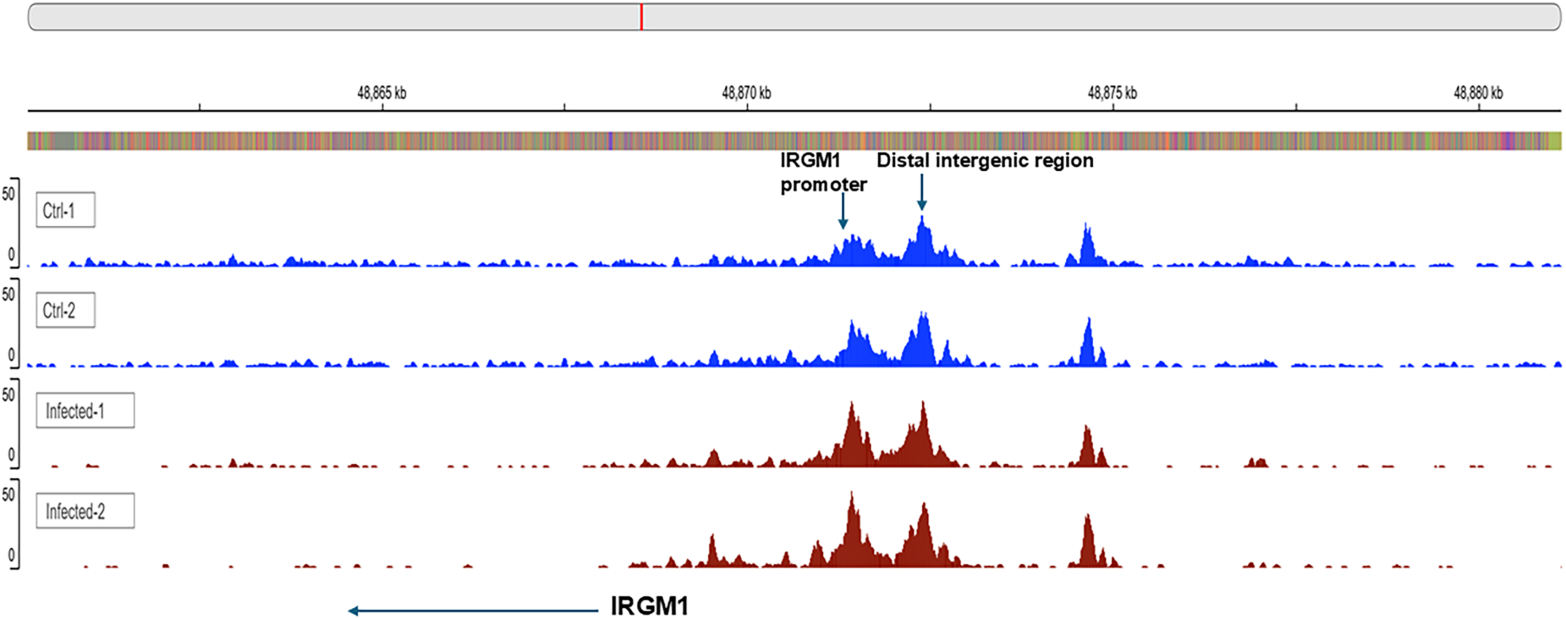
**Higher open chromatin near the IRGM1 promoter in spleen macrophages from latently infected mice.** Mice were ocularly infected with 2 × 10^5^ pfu/eye of HSV-1 or mock infected. On day 35 PI, spleens from infected mice were isolated, dissociated into single cell suspensions, and stained with the indicated antibodies. Lineage markers (Lin^−^); FITC-CD3, FITC-CD19, and FITC-Ly6g were used to exclude T cells, B cells, and neutrophils. Macrophages were identified as Lin⁻CD3⁻CD45⁺CD11b⁺F4/80⁺ cells, sorted by flow cytometry, and subjected to ATAC-seq to assess chromatin accessibility. Peaks of open chromatin were visualized by IGV web browser (UCSD). The *arrow* indicates an increase in open chromatin near the IRGM1 promoter.

To determine whether macrophages isolated from the spleens of latently infected mice exhibit a response similar to that of BM-derived macrophages described above, on day 35 PI, the spleens were harvested and dissociated to single cells, then stimulated with UV-inactivated HSV-1 strain McKrae (10 pfu/cell) for 24 hours. These cells were stained with antibodies against CD3, CD19, Ly6g, F4/80, CD45, and 7-AAD. The CD45^+^F4/80^+^CD3^−^CD19^−^Ly6g^−^ macrophages were sorted and subjected to ATAC-seq. Chromatin accessibility profiling revealed a pronounced increase in IRGM1 peak signal in macrophages from HSV-1-infected mice compared with those from mock infected controls, suggesting higher transcription of this gene in latently infected macrophages (Fig. 3, arrow, IRGM1). Similar to Figure 1, the second peak detected in both control and infected groups is located at the distal intergenic region to IRGM1 and has no peak elevation in contrast to the IRGM1 promoter region (see Fig. 3, arrow, distal intergenic region). Thus, our data suggest that IRGM1 plays a pivotal role in macrophage memory following HSV infection, potentially leading to a more effective response to recurrent HSV-1 infection.

### IRGM1^+^ Cells Are Upregulated in the Spleens of Latently Infected Mice Following Stimulation With HSV-1

**Figure 4.**
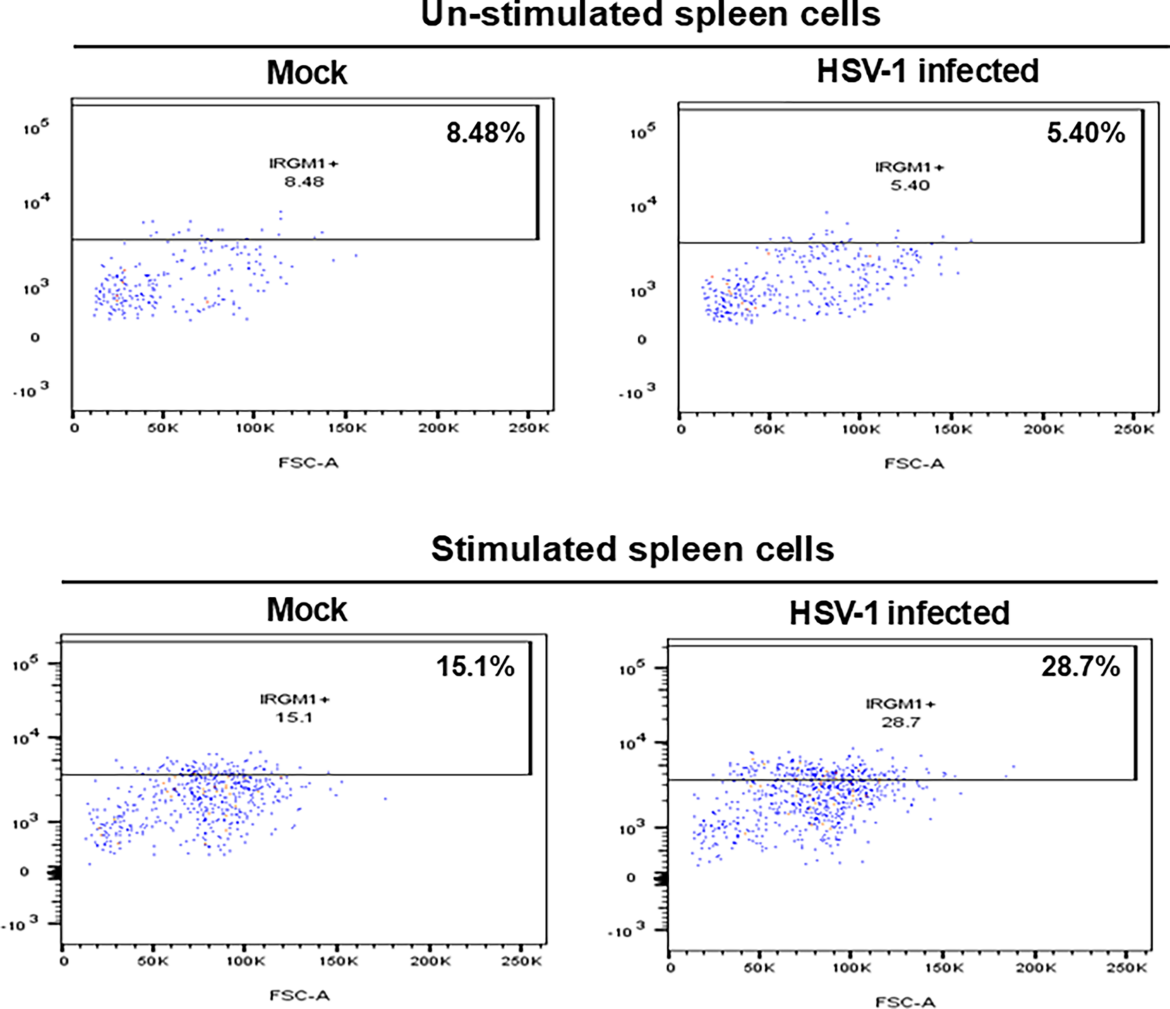
**Higher expression of IRGM1 in spleen macrophages from latently infected mice.** Mice were infected ocularly with HSV-1 or mock infected as described above. On day 35 PI, spleens were harvested and dissociated into single-cell suspensions, and red blood cells were removed. Cells were then plated and stimulated with UV-inactivated HSV-1 (10 pfu/cell) or mock stimulated for 24 hours. Cells were harvested and stained with the indicated antibodies, as described in Materials and Methods section. Stained cells were gated for CD45^+^F4/80^+^Lin^−^ macrophages, and the percentage of IRGM1^+^ cells was evaluated by flow cytometry. Data are represented as the percentage (%) of IRGM1^+^ cells in each group before and after stimulation.

Our ATAC analysis, as described above, suggests that macrophage memory is associated with IRGM1 upregulation. Thus, to directly determine whether IRGM1 is upregulated in macrophages isolated from latently infected mice, the mice were ocularly infected with 2 × 10^5^ pfu/eye of HSV-1 or mock-infected as above. On day 35 PI, the spleens were processed as described in Materials and Methods. Stained cells were gated for CD45^+^CD3^−^CD19^−^CD11C^−^Ly6G^−^F4/80^+^IRGM1^+^, and the percent of IRGM1^+^ cells was evaluated. In unstimulated samples, 8.48% of macrophages in the mock group and 5.40% in the HSV-1-infected group expressed IRGM1 (Fig. 4, unstimulated groups). In contrast, following UV-stimulation, IRGM1⁺ macrophages increased to 15.1% in the mock group and 28.7% in the latently infected group (see Fig. 4, stimulated groups). These results suggest that a greater number of IRGM1^+^ cells were present in the spleens of latently infected mice after stimulation than in the mock control group.

### IRGM1 Is Expressed in the Corneas of Infected Mice

**Figure 5.**
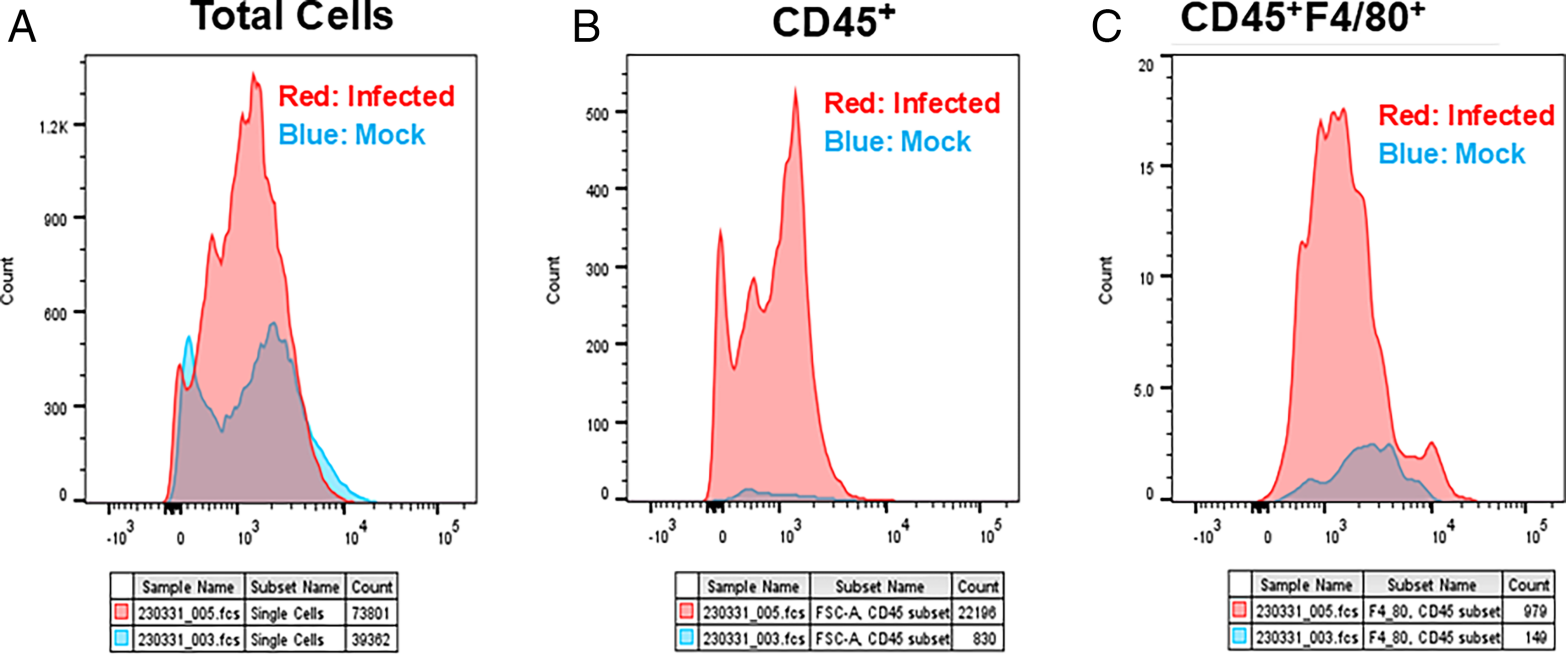
**Increased IRGM1 expression in the corneas of latently infected mice.** Mice were infected ocularly or mock infected as above. On day 35 PI, corneas were harvested and dissociated into single cell suspensions. Cells were then stained with antibodies against CD45, F4/80, and IRGM1. (**A**) The histogram of total IRGM1^+^ cells in the corneas of infected and mock infected mice. (**B**) Displays the histogram of CD45⁺IRGM1⁺ cells, whereas (**C**) shows the histogram of CD45⁺F4/80⁺IRGM1⁺ macrophages. *Blue* represents the histogram for the mock infected group, whereas *red* represents the histogram for the infected group. Sample sizes were 8 corneas for mock infected mice and 12 corneas for infected mice.

Similar to our data on BM and spleen macrophages described above (see Figs. 1^2^,^3^–4), we examined IRGM1 expression in latently infected corneas. Mice were infected ocularly as above, and corneas were harvested on day 35 PI, dissociated into single cells, and then stained with antibodies against CD45, F4/80, and IRGM1, as described in Materials and Methods. Mock infected mice served as controls. In Figure 5A, we analyzed the total corneal cell population in infected and mock infected mice. We observed a higher proportion of IRGM1⁺ cells in latently infected corneas compared with mock infected controls, as indicated by the shift in histogram peaks (see Fig. 5A, IRGM1^+^). In Figure 5B, we gated on CD45⁺ cells to assess IRGM1 expression in immune cells. Similar to the total population, the frequency of CD45⁺IRGM1⁺ cells were significantly higher in infected corneas compared to mock-infected controls (see Fig. 5B; CD45^+^IRGM1^+^). Finally, in Figure 5C, we further gated on the CD45⁺F4/80⁺IRGM1⁺ macrophage population to specifically examine IRGM1 expression in corneal macrophages. Consistent with earlier findings, IRGM1 expression in this gated macrophage subset was markedly higher in infected corneas compared to mock (see Fig. 5C; CD45⁺F4/80⁺IRGM1⁺). Collectively, these results demonstrate that IRGM1 is upregulated in corneal macrophages during latent HSV-1 infection, supporting the notion that corneal tissue-resident or infiltrating macrophages undergo trained immunity in response to HSV-1 exposure.

### IRGM1 Expression Is Upregulated in the TGs of Latently Infected Mice

**Figure 6.**
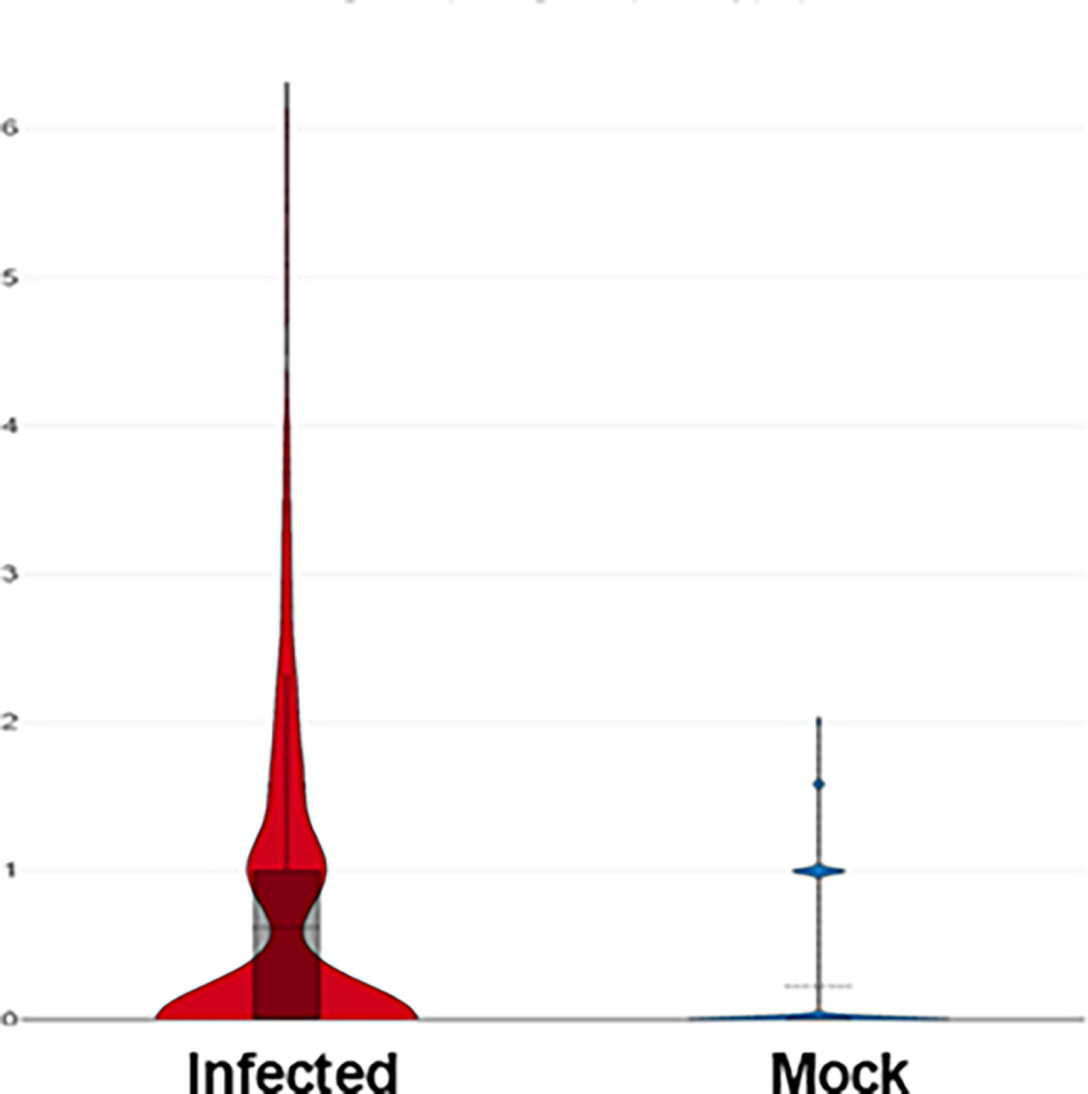
**IRGM1^+^ macrophages increase in TG of latently infected mice.** Mice were infected or mock infected as above. On day 35 PI, the TGs were harvested, pooled together (*n* = 30 TGs per group), and dissociated to single cell suspensions. Dead cells were removed using a dead cell removal kit, and CD45^+^ immune cells were isolated, as described in Materials and Methods section. IRGM1 expression in macrophages was analyzed, and data are presented as a violin plot of log^2^-transformed expression values. The mean IRGM1 expression in macrophages from infected mice was 0.59 compared to 0.22 in macrophages from mock infected mice.

Because the TG is the site of HSV-1 latency,^38^ we next investigated whether macrophages in the TGs of latently infected mice also express higher IRGM1. Thus, as in the above and in our previous studies,^30^ mice were infected ocularly with HSV-1 or mock-infected. On day 35 PI, the TGs were harvested, single cells were prepared, and CD45^+^ cells were sorted as described in Materials and Methods. We performed single cell sequencing on isolated CD45^+^ cells to determine the presence of IRGM1^+^ macrophages. As shown in Figure 6, the violin plot of IRGM1 expression was extracted from the single cell sequencing database. IRGM1 expression in the macrophages was significantly higher in the infected group compared to the mock group (see Fig. 6; mean value, infected 0.59 versus mock 0.22). These results suggest that, as in the BM, spleen, and cornea, higher expression of IRGM1+ cells was observed in the TGs of latently infected mice compared with the mock control group.

### IRGM1 Transcript Stays Upregulated on Day 70 PI in BM-Derived Macrophages, Spleen, Macrophages, and Total TGs of Latently Infected Mice

**Figure 7.**
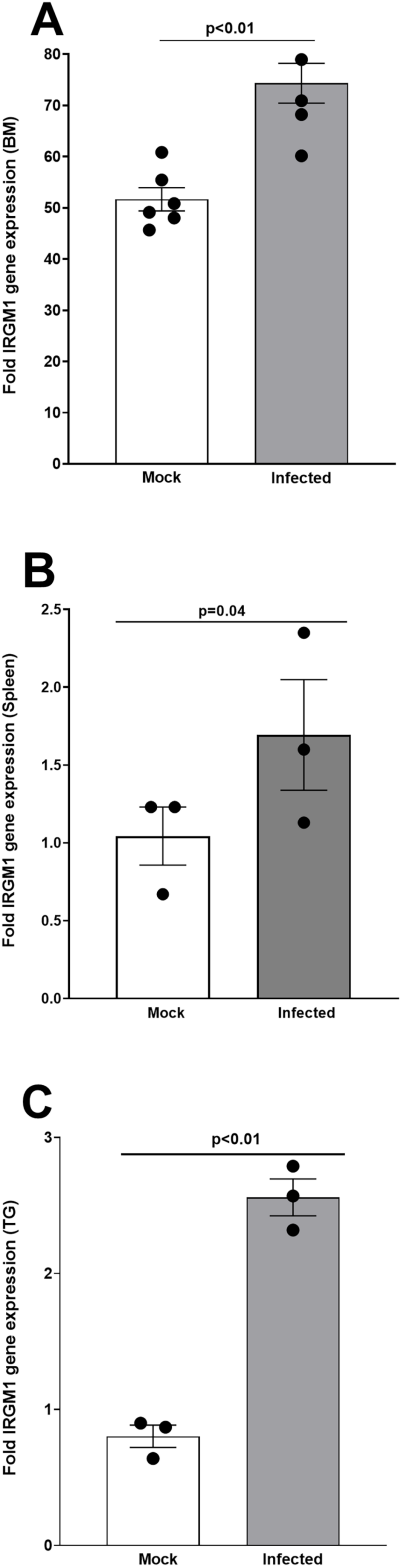
**Longevity of IRGM1 mRNA expression in BM, spleen macrophages, and TG of latently infected mice.** Mice were ocularly infected as described above, and on day 70 PI, mice were euthanized. RNA was isolated from infected BM-derived macrophages (**A**), isolated spleen macrophages (**B**), and total TG (**C**). Total RNA was used to measure IRGM1 expression by quantitative real-time PCR (qRT-PCR). RNA from naive mice was used as a baseline to calculate the relative expression of IRGM1 in each tissue. GAPDH expression was used to normalize relative expressions of IRGM1 transcript levels. Each bar represents the mean ± SEM from three mice.

Our ATAC, FACS, and single-cell analyses, as described above, suggested the importance of IRGM1 in macrophage memory responses to HSV-1 infection on day 35 PI. To determine whether this upregulation persists beyond day 35 PI, we evaluated IRGM1 transcript levels at day 70 PI, the final time point assessed in this study. Mice were ocularly infected with HSV-1 or mock infected as described above. On day 70 PI, the BM, spleens, and TG were harvested. BM- and spleen-derived macrophages were generated as described in Materials and Methods, and total TG tissue was processed for RNA extraction. For the spleen, macrophage populations were sorted and analyzed separately. BM-derived macrophages were stimulated with UV-inactivated HSV-1, and IRGM1 expression was assessed using quantitative RT-PCR. We observed a significant increase in IRGM1 expression in stimulated BM-derived macrophages from latently infected mice compared to the mock infected group (Fig. 7A; *P* < 0.01). Similarly, IRGM1 levels were significantly elevated in spleen-sorted macrophages from infected mice relative to those from mock infected controls (Fig. 7B; *P* = 0.04). Finally, IRGM1 transcript levels were markedly higher in the TG of latently infected mice compared with mock infected mice (Fig. 7C; *P* < 0.01). These results confirm that IRGM1 expression persists long-term in macrophages during HSV-1 latency, with elevated levels maintained at least through day 70 PI—the final time point assessed. This supports our conclusion that IRGM1 plays a sustained and pivotal role in macrophage memory (trained immunity) in response to HSV-1 infection.

## Discussion

The vertebrate immune system is divided into two primary components: innate and adaptive.^39^ Cells of the innate immune system recognize pathogens and tissue damage through germline-encoded pattern recognition receptors (PRRs).^40^ Engagement of PRRs is rapid and nonspecific, and includes responses such as phagocytosis, cell locomotion, killing of pathogens or cells, and cytokine production. In the context of ocular HSV-1 infection, activated macrophages are crucial for resolving geographical ulcers before they progress to corneal scarring.^19^^,^^41^ This is supported by the potential role of mononuclear phagocytes in degrading released macromolecules and scavenging dead cells in the cornea, while concomitantly mediating granulation tissue formation and remodeling.^4^ Our previous vaccine efficacy studies demonstrated that macrophages are essential for controlling HSV-1 replication and preventing ocular disease during primary infection in immunized mice. However, in the latent stage of infection, macrophages do not have any observable effect on latency-reactivation.^19^ Macrophages can destroy pathogens directly or indirectly via innate and adaptive immune responses, respectively.^42^ These studies highlighted the potential importance of macrophages in HSV-1 infection and underscored the need for further investigation into their diverse roles in infection and immunity.

Innate immune memory in macrophages has recently gained importance in providing protection in response to external stimuli and has been divided into tolerance and training.^12^^,^^24^ This concept holds promise with the emergence of immune memory in tissue-resident macrophages in vivo, such as microglia in the central nervous system.^24^ In bacterial infections, alveolar macrophages have demonstrated an effective memory response to infection, contributing to improved host defense upon re-exposure.^25^ Macrophages have a crucial role in the memory protection conferred by the BCG vaccine against *Mycobacterium tuberculosis* infection.^26^ Historically, immunological memory was considered an exclusive hallmark of adaptive immunity. However, mounting evidence indicates that innate immune cells and even specific tissue-resident stem cells can exhibit adaptive characteristics.^43^^–^^45^ Thus, as with adaptive immune memory, activation of the innate immune system can enhance responsiveness to subsequent antigenic stimuli. This process of “trained immunity” can protect a host against infection, but its potentially detrimental outcomes must also be considered. The term “immunological training” has been used as a salient marker for most immunopathological and infectious diseases. NK cells, DCs, B cells, ILC2, CD4^+^, and CD8^+^ cells have been shown to retain memory of infection,^46^^–^^54^ underscoring the broad relevance of trained immunity across immune cell types.

Our ATAC analysis demonstrated that macrophages from latently infected mice exhibit memory responses to HSV-1 infection, as evidenced by elevated IRGM1 expression in infected and stimulated cells relative to unstimulated infected cells. IRGM1 (also known as LRG-47) is a mouse orthologue of human IRGM, an intracellular protein of approximately 47 kDa and transcriptionally upregulated by the activation of IFN-γ.^55^^,^^56^ IRGM1^−/−^ mice have regular expression of myeloid and lymphoid cell populations but show reduced macrophage activation and increased susceptibility to infection by *Toxoplasma gondii*, *Listeria monocytogenes*, *Salmonella typhimurium*, and *Mycobacterium tuberculosis*, but not by murine cytomegalovirus (MCMV).^57^^–^^59^ IRGM1 regulates M1 macrophages motility with its expression and associated chromatin remodeling and histone modifications predominantly occurring during M1, rather than M2, macrophage activation.^60^ In addition, expression of the IRGM1 gene in M1 macrophages, rather than in M2 macrophages, and chromatin remodeling, along with histone modifications, occur during M1 macrophage activation.^61^ Immunity-Related GTPase Family M Member 1(IRGM1) plays a crucial protective role during HSV-1 infection. Upon viral invasion, interferon signaling induces IRGM1 expression, enhancing the cell's autophagic capacity.^62^^,^^63^ By promoting autophagosome formation and lysosomal degradation, IRGM1 facilitates the clearance of viral components and helps maintain cellular homeostasis, thereby limiting HSV-1 replication. However, HSV-1 has evolved countermeasures to evade this defense: its neurovirulence protein ICP34.5 binds to Beclin-1, a central regulator of autophagy, effectively blocking autophagy and antagonizing IRGM1’s antiviral function.^64^^,^^65^ In addition to its role in restricting viral replication, IRGM1 also contributes to the regulation of neuroinflammation, preserves mitochondrial integrity, and prevents virus-induced neuronal death.^66^ Together, these functions highlight IRGM1 as a key mediator of host resistance and cellular resilience during HSV-1 infection. Our data indicate that IRGM1 memory responds robustly to virus stimulation, suggesting its critical role in macrophage training against HSV-1, consistent with a group studying the role of human IRGM in viral infections.^66^ Consistent with the ATAC-seq and flow cytometry findings in BM and spleen-derived macrophages, corneal macrophages from latently infected mice also showed significantly higher IRGM1 expression compared with mock infected controls. A similar pattern was observed in the TG, a key site of HSV-1 latency. We also tested the durability of IRGM1 memory responses by extending the duration of infection in our in vivo model. Infected tissues at day 70 PI still showed elevated IRGM1 expression compared with controls, demonstrating the persistence of macrophage memory over time.

Therefore, our study provides novel evidence that trained macrophages, marked by IRGM1 expression, persist during HSV-1 latency. Absence of IRGM1 in macrophages leads to enhanced pro-inflammatory cytokines, thus causing inflammation by metabolic alterations in macrophage functions.^30^ Overall, the control of homeostasis and cytokine production in macrophages is thought to be the primary mechanisms by which IRGM/IRGM1 regulate inflammatory diseases.^67^ Our results suggest that macrophages retain memory of HSV-1 infection, and this memory may contribute to enhanced protection against ocular HSV-1 infection. Overall, our findings indicate that macrophages retain a trained immunity to HSV-1 infection, which may contribute to enhanced protection against ocular disease caused by this virus. This study advances the understanding of macrophage-trained immunity in ocular HSV-1 infection and highlights IRGM1 as a potential therapeutic target. Exploring macrophage memory could pave the way for innovative strategies to prevent HSV-1 reactivation and subsequent ocular disease.
